# Fiscal rules, powerful levers for controlling the health budget? Evidence from 32 OECD countries

**DOI:** 10.1186/s12889-018-5198-y

**Published:** 2018-03-01

**Authors:** Herman Christiaan Schakel, Erilia Hao Wu, Patrick Jeurissen

**Affiliations:** 1 Celsus, Academy for Sustainable Healthcare, IQ Healthcare, Radboud University and Medical Center, P.O. Box 9101, 114 6500 HB Nijmegen, The Netherlands; 2 NYU Wagner School of Public Service, New York University, 295 Lafayette street, New York, NY 10012 USA

**Keywords:** Health budgeting, Fiscal rules, Budgetary governance, OECD

## Abstract

**Background:**

Publicly funded healthcare forms an intricate part of government spending in most Organisation for Economic Co-operation and Development (OECD) countries, because of its reliance on entitlements and dedicated revenue streams. The impact of budgetary rules and procedures on publicly funded health care might thus be different from other spending categories. In this study we focus on the potential of fiscal rules to contain these costs and their design features.

**Methods:**

We assess the relationship between fiscal rules and the level of public health care expenditure of 32 (OECD) countries between 1985 and 2014. Our dataset consists of health care expenditure data of the OECD and data on fiscal rules of the International Monetary Fund (IMF) for that same period. Through a multivariate regression analysis, we estimate the association between fiscal rules and its subcategories and inflation adjusted public health care expenditure. We control for population, Gross Domestic Product (GDP), debt and whether countries received an IMF bailout for the specific period. In all our regressions we include country and year fixed effects.

**Results:**

The presence of a fiscal rule on average is associated with a 3 % reduction of public health care expenditure. Supranational balanced budget rules are associated with some 8 % lower expenditure. Health service provision-oriented countries with more passive purchasing structures seem less capable of containing costs through fiscal rules. Fiscal rules demonstrate lagged effectiveness; the potential for expenditure reduction increases after one and two years of fiscal rule implementation. Finally, we find evidence that fiscal frameworks that incorporate multi-year expenditure ceilings show additional potential for cost control.

**Conclusions:**

Our study shows that there seems a clear relationship between the potential of fiscal rules and budgeting health expenses. Using fiscal rules to contain the level of health care expenditure can thus be a necessary precondition for successful strategies for cost control.

## Background

There have been widespread efforts to identify the main drivers behind the growth of health care expenditure [1–4], as identifying these drivers allows policy makers to develop (effective) measures to contain health care costs. Important determinants in these studies include demographic changes such as ageing societies, income and price developments and the spread of medical technologies. The relation between health expenditure and the Gross Domestic Product (GDP) is most prominent throughout the literature, as it captures many underlying economic and societal developments. Institutional and regulatory determinants such as the level of debt cautiously point to an inverse relationship; as debt levels rise, public health care expenditure seems to decline [1].

Policy measures that target the drivers of health expenditure growth have been much debated over the past decades. Much less prolific has been the literature on the impact of budgetary governance, or the use of fiscal rules in particular, on offsetting the rise of health care costs for the medium to long-term. Over the past decade, governments of Organisation for Economic Co-operation and Development (OECD) countries have increasingly devised fiscal frameworks to contain government spending [5]. Yet few studies examine the effectiveness of fiscal frameworks for such public health care expenditure. Reeves et al. [6] point out that health sectors are not protected during periods of austerity, thereby suggesting that fiscal frameworks might be successful in containing health budgets. It is thus of interest to study the potential of fiscal rules to contain publicly funded health care, and its design features.

Throughout the OECD, public health expenses form an intricate part of government spending. On the one hand they highly correlate with economic growth. This is illustrated by the notable slowdown of health expenditure in the aftermath of the economic and financial crisis of 2008. On the other hand, cutting back on health entitlements or provisions is often politically fraught and unpopular amongst politicians, propelling them towards cutbacks in other areas, such as discretionary non-health spending [7]. There are both exogenous and health care specific circumstances that impact expenditure development, which are not present in most other public spending categories.

It is thus of interest, to study the fiscal rules that govern the decision making process on health expenditure and to ascertain what their influence has been on the level of publicly funded health budgets in recent decades. To this end, we compare expenditure data of publicly funded health care of 32 OECD countries between 1985 and 2014, with a dataset on fiscal rules in those countries by the IMF for that same period. Our analysis covers changes in budget policies from year to year and the results thus provide a comparison between different countries and within a single country.

The International Monetary Fund (IMF) defines a fiscal rule as a rule that imposes a long-lasting constraint on fiscal policy through numerical limits on budgetary aggregates [5]. A considerable body of literature in the field of public finance discusses the relationship between fiscal rules and fiscal performance. Its main findings are that ‘tighter and more encompassing rules are correlated with stronger cyclically adjusted primary balances in EU countries, that balanced budget rules and debt rules have a greater impact on budgetary outcomes than expenditure rules and revenue rules, and that rules that cover wider levels of government are associated with stronger fiscal discipline’ [5].

The IMF identifies four categories of fiscal rules: expenditure rules (ER), revenue rules (RR), debt rules (DR) and balanced budget rules (BBR).^1^ Our focus is on ERs and BBRs, because they impact expenditure levels more directly than the other two. For BBRs we make a distinction between national and supranational rules and countries that operate both in tandem. The latter is particularly relevant for EU countries, as BBRs are part of the budgetary framework of the 1992 Maastricht Treaty.

We assess if fiscal rules have a more limited impact on countries that are health service provision-oriented (passive purchasing that limits countervailing powers towards providers) and rely on social insurance contributions that underline the entitlement character of health care services [24]. We hypothesize that such systems are less impacted by fiscal policy. Because cost-control policies in healthcare take time to implement, we are additionally interested if rules become more effective one and two years after implementation of such a rule. In addition to the direct effect of fiscal rules, we look at the impact of enforcement and monitoring mechanisms that often accompany them, since evidence suggests that expanding fiscal rules with these mechanisms increases their effectiveness.

Fiscal rules aim to depoliticize policymaking. By removing discretionary intervention, they seek to achieve predictability of government action [8]. Over the past decades, fiscal rules have been relatively successful in containing government expenditure [9, 10]. Guichard et al. [11] find that fiscal rules with embedded expenditure targets have been associated with larger fiscal consolidations. At the same time, the impact of fiscal rules seems context dependent. Dahan and Strawczynski [12] for example find a negative effect of fiscal rules on the ratio of social transfers to government consumption. In general, the authors point out, government consumption seems more resistant to the pressures of fiscal rules than social transfers. If however, a country shows a strong legal commitment for a social safety net, this effect disappears. They draw the conclusion that governments, when designing fiscal policies, should take into account the effect of fiscal rules on spending composition.

The potential impact of fiscal rules on government spending seems well established. So what are important design features of fiscal rules? Most scholars agree that the existence of rules is no guarantee in itself for fiscal prudence. In order for rules to be successful, they have to be well designed and there has to be political willingness to comply [13–15]. This willingness can be codified at various institutional levels: legal bases for fiscal rules can vary from constitutional, to statutory, and coalition or political agreement. With some caution, evidence shows that a strong legal basis and strict enforcement seem to have had a beneficial impact on fiscal performance in the past decades throughout the OECD [5]. The exact interaction between fiscal discipline and fiscal rules is however complicated, since countries with strong fiscal discipline do not necessarily dispose of fiscal rules, and countries with such rules that do not observe or renew them do not necessarily demonstrate fiscal discipline [16]. This links to possible omitted variable bias: the correlation between strong fiscal performance and the use of fiscal rules may in fact be the result of political commitment rather than the existence of a rule.

Schick [17] points to the importance of a budget horizon of several years. He argues that the annual budget process is ‘an invitation to evasion’. By introducing a budget horizon that covers several budget years the propensity to hold off difficult policy measures is limited, since governments are compelled to bring their policies in line with the fiscal rule [5].

Another important element seems to ‘empower independent overseers to review budget actions and to point out actual or potential violations’ [17]. In addition, independent bodies that both set budget assumptions and monitor the implementation of budgetary measures potentially strengthen the fiscal framework. These activities are often performed by economic scoring agencies or independent fiscal bodies that are increasingly active throughout the OECD [8]. Debrun and Kumar [15] provide some evidence that these institutions contribute to budgetary outcomes, although they do also point to the possibility of reverse causality, in which countries ‘lock in’ already existing fiscal consolidation preferences.

The literature on the association between fiscal policy and health care expenditure is limited. Most contributions focus on how budgetary governance structures impact decisions on health. White [18] for example discusses how budget professionals and health policy makers collide in respect to the health budget. He argues that the former often have the upper hand, concluding that political forces are in the end stronger than the budget or health professionals. This observation is shared by Schakel et al. [19], who provide a qualitative analysis of the actual use of fiscal rules in the Netherlands and the United States. Their conclusion is that fiscal rules seem to have more bearing on budgetary outcomes than on the budget process itself, in other words: the numbers prevail the compliance with the budgetary process.

An empirical study of the cost cutting potential of fiscal rules in respect to public health expenditure seems relevant for a number of reasons. First, the health budget of OECD countries has risen faster over the past decades than other spending categories and GDP [7]. It thus seems of interest to study what the impact of fiscal rules has been on expenditure development. Second, as we have seen above, the effectiveness of fiscal rules as a depoliticized policy framework is under debate. This may be even more so in the case of the health budget: health care is often seen as a ‘right’ and is labeled as ‘high priority’ by citizens. Thirdly, controlling the health budget requires effective coordination between many stakeholders which often creates time lags and information shortages. Steering costs effectively using fiscal rules therefore seems challenging.

## Methods

### Data

We gather data on health expenditure of 32 OECD countries between 1985 and 2014 from the OECD health statistics database [20]. OECD member countries Turkey and Korea are not included in this analysis, due to a lack of IMF data on fiscal rules for these countries. Of these 32 countries, 21 are part of the European Union and thus subject to the ‘corrective arm’ as part of the Stability and Growth Pact (SGP) of the EU.^2^ This means that for these countries, a supranational balanced budget rule has been in place since the introduction of the SGP (1992), or as soon as a country became a member of the Union.

From the database we derive the annual nominal expenditure figures in international dollar terms, which we adjust for purchasing power parity that is based on year 2005.

Next, we search the IMF database on fiscal rules for the same period [21]. The database includes about 70 variables on fiscal rules and comprises quantitative and qualitative information on various characteristics, such as the number and type of rules, legal basis, coverage, escape clauses, enforcement, and supporting procedures. The dataset is not specifically catered towards the health budget. For this reason, we correct our dataset for those countries that have excluded health expenditure from their general fiscal framework for a given period, which in our timeframe, include Austria, Switzerland and the United States. Table 1 in Appendix 2 provides an overview of the active fiscal rules of our 32-country sample between 1985 and 2014.

**Table 1:** The association between fiscal rules and the level of public health care expenditure

	Public health care expenditure in log						
	(1)	(2)	(3)	(4)	(5)	(6)	(7)
	32 OECD countries				Exclude Cluster I countries		
FR	−0.03**				− 0.04***		
	(0.01)				(0.01)		
ER		−0.04**				−0.05***	
		(0.01)				(0.01)	
BBR			−0.04**				−0.05***
			(0.01)				(0.01)
BBR (detailed levels)							
National				−0.02			
				(0.02)			
Supranational				−0.06***			
				(0.02)			
Both				−0.08***			
				(0.02)			
*Country FE*	Y	Y	Y	Y	Y	Y	Y
*Year FE*	Y	Y	Y	Y	Y	Y	Y
*N*	717	717	717	717	609	609	609
adj. *R*^2^	0.997	0.997	0.997	0.997	0.997	0.997	0.997

Columns 1 to 4 include all countries in the dataset, and column 5 to 7 exclude cluster I countries. Dependent variable public health care expenditure is adjusted for inflation and purchasing power. Appendix 3 provides a full table, including all control variables

Standard errors are in parentheses

**p < 0.01, ***p < 0.001

### Estimating the effect of fiscal rules on public health care expenditure

The aim of this study is to estimate the following equation:

1 $$ {Expenditure}_{i,t}={\beta}_0+{\beta}_1F{R}_{i,t}+{X}_{i,t}\gamma +{\alpha}_1\sum countr{y}_i+{\alpha}_2\sum yea{r}_t+{\epsilon}_{i,t} $$

Our primary independent variable of interest, *fiscal rules* (FR), is presented as a dummy variable. We study the overall presence of a fiscal rule and further discriminate between expenditure rules and balance budget rules since those seem most relevant for the direct steering of health expenses.

The dependent variable *expenditure*_*i*, *t*_ measures the inflation adjusted public health care expenditure of country *i* in year *t*. *X*_*i*, *t*_ represents a vector of control variables, which includes several demographic and economic characteristics. In particular, we use four control variables. First, *GDP*, which serves as a proxy for both income and price development. Some national longitudinal studies suggest an income elasticity slightly greater than one, indicating that income and health expenditure are highly correlated [3]. Second, we use *debt* to indicate the level of public debt, as governments with higher debts are more susceptible to cutting back on the budget, including on health entitlements and provisions. Third, the overall *population*.^3^ Fourth, a dummy variable for which an *IMF bailout* loan was received in country *i* during year *t*. The IMF coerces grantees to implement structural reforms to address institutional or economic weaknesses, in addition to policies that maintain macroeconomic stability. All control variables are explained at greater details in Appendix 1.

Finally, because of the non-random assignment of the fiscal rules and the large variances in both the external environment (such as the impact of the global economy), as well as the internal characteristics (such as country-specific attitudes towards health care consumption) that may not have been captured in the current model, we add country and year fixed effects in all our regressions.

It is generally assumed that health systems that rely on entitlements and dedicated revenue streams face greater budgetary challenges [7, 18, 22, 23]. For this reason we run a separate regression that excludes five countries whose health entitlements are based on contributions, and share other features such as a high share of public funding as a percentage of total health expenditure and low levels of regulation as well as passive purchasing structures [24]. This cluster is composed of Austria, Belgium, Germany, France and Luxemburg^4^ (‘Cluster I countries’). Other features that set these countries apart from other categories, is a high level of autonomy of self-employed doctors and a high level of freedom of consumer choice. Cluster II and Cluster III countries (Great Britain, Sweden, Portugal, among others) on the other hand have more control over access to medical care and the salaries of GPs and specialists. There is thus reason to assume that ‘Cluster I countries’ have fewer levers to control costs, and therefore might be less impacted by fiscal rules.

### Possible lagged effectiveness of fiscal rules

It is possible that the cost cutting potential of fiscal rules increases when a time lag is introduced. There are two main reasons to assume this effect is present. First, a lag in reporting on health expenditure and a subsequent delay in the implementation of containment measures is widely observed throughout the OECD.^5^ The OECD suggests that this delay is often due to data-collection issues or reporting from health care institutions/insurers or sub-national governments. It additionally notes that: ‘delays in information made it harder for them [budget officials] to work with Health Ministries to take corrective measures through the year and in some cases prompt additional savings within a short time frame to meet end-of-year fiscal objectives’ [7]. The second reason is that modifications to the benefit basket or the level of out-of-pocket costs in the current year, two strategies that are often sought for by policymakers, are difficult to attain. Under these circumstances, introducing such modifications in year *t* will not lead to immediate cut backs on the budget, but at the earliest in year *t + 1*. We therefore introduce a two-tailed one- or two-year time lag: both lagged implementation and persisting effectiveness (after a rule has been suspended) in one or two years.

We re-write Eq. (1) for such time lag in Eq. (2):

2 $$ {Expenditure}_{i,t}={\beta}_0+{\beta}_1F{R}_{i,t-k}+{X}_{i,t}\gamma +{\alpha}_1\sum countr{y}_i+{\alpha}_2\sum yea{r}_t+{\epsilon}_{i,t} $$

Where *k* represents the number of years, which subsequently equals to 1 or 2.

### The impact of further supporting procedures

Enforcement and monitoring mechanisms in theory provide additional safeguards that fiscal rules will be complied with. According to Budina et al. [5], these features popularized throughout advanced and emerging economies since the onset of the financial crisis of 2008. The database provides a subset of data that describe several of these supporting procedures. We incorporate such variables as Eq. (3):

3 $$ {Expenditure}_{i,t}={\beta}_0+{\beta}_1F{R}_{i,t}+{\beta}_2F{R}_{i,t}\times supportin{g}_{procedure{s}_{i,t}}+{\beta}_3 supportin{g}_{i,t}+{X}_{i,t}\gamma +{\alpha}_1\sum countr{y}_i+{\alpha}_2\sum yea{r}_t+{\epsilon}_{i,t} $$

*Supporting_procedures* represents one of the following variables in each of our models: 1) the legal basis of a fiscal rule (level 1 = political commitment or coalition agreement, level 2 = statutory or constitutional), 2) the existence of multi-year expenditure ceilings, 3) an independent body that sets budgetary assumptions and 4) an independent body monitoring budget implementation. The coefficient *β*_2_ indicates the additional effect of a particular supporting procedure on health care expenditure. It is important to note that the IMF database is constructed in such a way that for some of these supporting procedures, they only exist when a fiscal rule is present, which is why in our results, *β*_3_ in some models were not estimated.

## Results

**Fig. 1:**
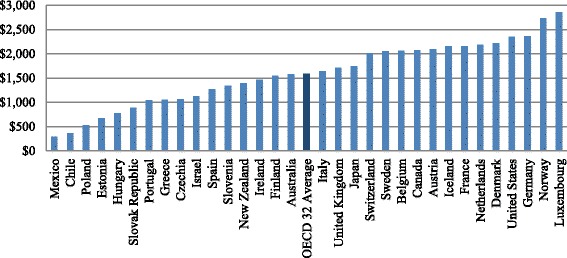
Per Capita Public Health Care Expenditure OECD 32 Average 1985–2014^*^. ^*^Source: Adapted from OECD 2016 (Per capita, constant prices, constant PPPs, OECD base year)

Figure 1 provides an overview of the per capita average public health care spending between 1985 and 2014. In Appendix 1 we provide a more detailed explanation of what constitutes OECD’s definition of public health care expenditure.

### The effects of fiscal rules on public health care expenditure

Table 1 provides an overview of the association between fiscal rules and the level of public health care expenditure, for both the 32 OECD countries and excluding the ‘Cluster I countries’.

The presence of a FR on average is associated with 3% decrease in health care expenditure at a 1% alpha level. Broken down by types of rules, ER demonstrates a greater potential for expenditure reduction at 4%. Overall, countries with a BBR on average devote 4% less of their public resources to health care. However, its influence deserves further elaboration: a national level BBR reveals a statistically insignificant negative relationship with health care expenditure; yet, when this is adopted at a supranational level, or at both levels combined, the effect becomes considerably significant, at 6 and 8% respectively. This suggests that for EU countries, efforts by the European Commission to enforce a 3% maximum deficit seem to have a significant effect on health expenses, one of the largest outlays within the central budget. This effect might become even stronger as a result of the increasing attention of health reforms within the framework of the European Semester process [25].

When excluding ‘Cluster I countries’ from the regression, we find that coefficients of FR, ER and BBR increase by one percentage point, respectively. This suggests that countries that are health services provision-oriented, seem indeed less capable of controlling public health care expenditure through fiscal rules, than other countries.

**Fig. 2:**
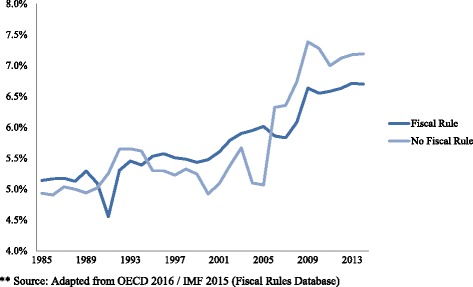
Publicly funded health care expenditure (as % GDP) OECD 32 pooled by presence of a fiscal rule**

Figure 2 presents publicly funded health care expenditure as percentage of GDP, pooled by country-years with and without fiscal rules. It indicates that on average, budgetary frameworks that incorporate fiscal rules show lower health care expenditure growth. Especially after the financial crisis of 2008, stricter budgetary frameworks are associated with lower expenditure growth. Although the figure does not control for variables that are included in the model, it does suggest that over time, governments do benefit from stricter budgetary frameworks. The figure might suggest that the regression coefficient we find in our main model is underestimated; the cost cutting potential of fiscal rules, especially in post crisis years, seems considerable. In fact, in column 1 of Appendix 6, in which we replaced the outcome variable with public healthcare expenditure as a percentage of GDP, we see the presence of FRs is associated with − 40% decrease in the outcome. It suggests that countries with relative higher shares of health care spending seem more successful in exerting fiscal pressure than countries with lower shares of spending. A sensitivity analysis, in which we replace some key variables of our main model (columns 2–4 of Appendix 6), however confirms the robustness of our initial findings and the coefficient of our independent variable of interest.

### The possible lagged effect of fiscal rules on public health care expenditure

Table 2 highlights the lagged effectiveness of fiscal rules. The association between overall FR and ER and the health budget increases by one percentage point for each additional year after a government starts implementation. At the same time, we find no such effects with BBR. All of the models described above yield statistical significance on our primary independent variable of interest. These findings suggest that the cost cutting potential of fiscal rules becomes greater after one and two years.

**Table 2:** The lagged effectiveness of fiscal rules

	Public health care expenditure in log					
	(1)	(2)	(3)	(4)	(5)	(6)
	One year lag			Two year lag		
FR	−0.04***			−0.05***		
	(0.01)			(0.01)		
ER		−0.05***			−0.06***	
		(0.01)			(0.01)	
BBR			−0.04***			−0.05***
			(0.01)			(0.01)
*Country FE*	Y	Y	Y	Y	Y	Y
*Year FE*	Y	Y	Y	Y	Y	Y
*N*	717	717	717	717	717	717
adj. *R*^2^	0.997	0.997	0.997	0.997	0.997	0.997

Columns 1 to 3 show effects with one year lag, and columns 4 to 6 show effects with two year lag. Dependent variable public health care expenditure is adjusted for inflation and purchasing power. Appendix 4 provides a full table, including all control variables. Standard errors are in parentheses

***p < 0.001

### The additional effects of supporting procedures

The results in Table 3 suggest that adopting supporting procedures has to some extent an additional limiting effect on public health care expenditure, even though the magnitude of such effects is mixed.

**Table 3:** The additional impact on health care expenditure from supporting procedures

	Public health care expenditure in log				
	(1)	(2)	(3)	(4)	(5)
	ER	BBR		FR	
FR	0.09	−0.03*	−0.02	−0.03**	−0.03*
	(0.05)	(0.01)	(0.01)	(0.01)	(0.01)
FR×FR legal basis Level 1	−0.17***	− 0.06***			
	(0.04)	(0.02)			
Level 2	0.17	0.04*			
	(0.10)	(0.02)			
FR × budget ceiling			−0.05***		
			(0.01)		
FR × independent body setting				0.01	
				(0.02)	
FR × independent monitoring					0.06
					(0.06)
*Country FE*	Y	Y	Y	Y	Y
*Year FE*	Y	Y	Y	Y	Y
*N*	717	717	717	717	717
adj. *R*^2^	0.997	0.997	0.997	0.997	0.997

Independent variable FR is presented in different forms. In columns 1 and 2, it is presented as ER and BBR, respectively, where legal basis level 1 indicates either a political commitment or coalition agreement, level 2 indicates either a statutory and constitutional level enforcement. In columns 3 to 5, it is presented as aggregate FR. Appendix 5 provides a full table, including all control variables. Standard errors are in parentheses

*p < 0.05, **p < 0.01, ***p < 0.001

As columns 3–5 suggest, of all the studied enforcement and monitoring mechanisms, the existence of a fiscal rule in combination with a strict multi-year expenditure ceiling shows the most promise in terms of cost containment, at approximately 7% reduction. The presence of an independent body setting budgetary assumptions or an independent body monitoring budget implementation yields no additional effect at statistical significant levels. The coefficients of each interaction terms and the primary independent variable of interest are jointly significant. However, as such enforcement and monitoring procedures are closely correlated with the presence of a fiscal rule, the true magnitude of their impacts is worth further investigation.

The distinction between different levels of legal frameworks for public health care expenditure seems relevant. An ER supported by a legal framework has a limiting effect on the level of public health expenditure. Interestingly, political frameworks (legal basis level 1) are much more effective than those that are enforced by legal rules (statutory or constitutional). For both ERs and BBRs, it seems that political frameworks correlate with lower levels of expenditure than ones that are enshrined in statutes or the constitution. Especially, expenditure rules that are underwritten by political goals seem effective versus other frameworks.

## Discussion

The main goal of this paper is to establish whether governments that use fiscal rules – on average and ceteris paribus – are better equipped to contain health care expenditure than governments without such rules. However, given that the level of spending dedicated to health care ultimately reflects a political choice, it is also conceivable that some countries willingly devote a larger share of resources to health care than other countries do, while at the same time complying with existing rules and fiscal frameworks. Our analysis does not reveal this relationship – which should be an analysis with budgetary overruns as the dependent variable – but instead describes the *potential* of fiscal rules in relation to budgeting health expenses. This study shows that such a potential seems clearly present. The cost cutting potential of fiscal rules still seems relatively modest and compares to the association between a country’s debt and that same budget (see Appendix 3 for details). On average, the association between having a fiscal rule in place and the per capita decrease in health spending translates to roughly $ 100.-. In line with the literature on the determinants of the growth of health expenditure, GDP explains most of its variance. The relation between the relative share of publicly funded health care and the cost cutting potential of fiscal rules seems present. Further research in this area could potentially demonstrate at what cutoff fiscal pressures are most effective.

In a substantial number of OECD countries health care is a sub-central responsibility, while sub-central fiscal policy is commonly an integral part of general government fiscal policy. Some evidence suggests that widespread decentralization of health systems has often increased health spending over the past decades [26]. Sub-central fiscal policies mostly focus on borrowing and debt rules, and less on expenditure. Moreover it seems that sub-central fiscal policies are often redundant due to the high level of discretion of the general government in addressing sub-central fiscal challenges [27]. For this reason, we do not expand on the role sub-central governments play in controlling health budgets. We hypothesize that sub-central decision making on public health expenditure will align with fiscal rules that govern the decision making of the general government.

Much of our results are in line with the more qualitative body of evidence that exists on budgeting for health emanating from OECD surveys, country studies and international comparisons. A quantitative multi-level study by Getzen [28] shows that at the highest levels of decision-making, budget constraints are determinative for the amount of resources allocated, rather than “the amount of disease”. Our results seem to align with these findings.

One area of future research could be to examine the association between fiscal rules and various health care utilization categories. A similar regression with average length of stay in a curative care facility as the dependent variable shows a positive correlation with having a fiscal rule in place (see Appendix 7). This suggests that budgetary restrictions do not result in shorter hospital admissions, which could in turn point to net efficiency gains. A next step would be to determine if these efficiency gains translate into lower prices or volumes of care.

Our results further suggest that budgetary governance for health has been most successful within the fiscal framework of the European Union. We cannot ascertain the extent to which supranational BBRs have enforced structural health reforms or simply enabled budget cuts. Greer et al. [29] find that, specific for the EU, ‘generic calls for reorganization are having less effect than cruder, more direct policies to reduce expenditure such as reduction in access, reduction in pay, or less expenditure’. Irrespective of the longer-term impact of fiscal policy, we can cautiously draw the conclusion that Supranational Balanced Budget Rules have shown substantial potential for cost containment of health budgets.

Another finding is that the specific design features of health systems seem to correlate with the susceptibility of fiscal pressures. Although this may seem intuitive, it seems of interest to acknowledge the interaction between fiscal policy and health systems design. Largely decentralized systems (such as Switzerland or Italy) are in many ways different from more centralized health systems [30, 31]. The same goes for health systems that operate as bureaus (such as the United Kingdom) compared to those that run entitlement programs (such as the Netherlands). These health systems each face different challenges in terms of fiscal sustainability and viable enforcement mechanisms and operate distinct budgetary control procedures [22, 23, 32]. The impact of health systems on health expenses is discussed extensively in the literature [33, 34], although clear relations are difficult to find [35]. When excluding ‘Cluster I countries’ from the regression, we have seen that the cost cutting potential of fiscal rules increases. It could be that the FRs within ‘Cluster I countries’ apply less stringent fiscal policies with regards to overspending. In any case, interactions between health systems design and fiscal frameworks seem to exist. Further research on this specific interaction seems warranted.

## Conclusions

Our results can be seen as a call for attention to budgetary governance, which turns out to be a critical feature in studying the success of different systems in terms of fiscal performance, and the assessment of preconditions that determine such success. A recent OECD study aims to explain the drivers of public health expenditure by assessing policy and institutional variables [36]. The main finding is that policy and institutional variables explain some 23% of public health expenditures. Our study supports such findings with respect to fiscal institutions.

The association between fiscal rules and a decrease of the health budget becomes stronger one and two years after implementation. This provides noteworthy policy implications. Budget (preparation) cycles are generally on an annual basis, while our results suggest that budgetary recoups often cannot easily take place within this short timeframe. It seems crucial for policy makers and budget officials to acknowledge these parallel realities and model their budgetary frameworks accordingly. Thus it is not very surprising that multi-year expenditure ceilings further enhance the possible impact of fiscal rules.

Finally, we find that the shape of the legal frameworks additionally impacts the potential to limit the health budget; politically constructed frameworks are more strongly associated with lower levels of health expenditures than rules that are confined in legislation. This finding requires further investigation but might align with Schick’s statement that a rule is as strong as the underlying political will to enforce it.

## Appendix 1

**Table 4:** Description of variables

Variable	Definition	Data Source
Public Health Care Expenditure	Health expenditure incurred by public funds. Public funds are state, regional and local Government bodies and social security schemes. Public capital formation on health includes publicly financed investment in health facilities plus capital transfers to the private sector for hospital construction and equipment.	OECD 2015
Debt	General government debt is the amount of a country’s total gross government debt. It is an indicator of an economy’s budgetary health and a key factor for the sustainability of government finance. *Debt* is commonly defined as a specific subset of liabilities identified according to the types of financial instruments included or excluded. Debt is thus obtained as the sum of the following liability categories (as applicable): currency and deposits; securities other than shares, except financial derivatives; loans; insurance technical reserves; and other accounts payable. Changes in government debt over time reflect the impact of government deficits. This indicator is measured as a percentage of GDP. Data are under System of National Accounts (SNA 1993) for all countries except for Australia and United States (SNA 2008). Studies by Pammolli et al. and Gerdtham et al. [1, 32] discuss the relation between debt and public health expenditure and argue that countries with higher debt exhibit lower level of public expenditure.	OECD 2015
GDP	Gross domestic product is an aggregate measure of production equal to the sum of the gross values added of all resident institutional units engaged in production (plus any taxes, and minus any subsidies, on products not included in the value of their outputs). The sum of the final uses of goods and services (all uses except intermediate consumption) measured in purchasers’ prices, less the value of imports of goods and services, or the sum of primary incomes distributed by resident producer units. We have included this variable – supported by the literature on the determinants of health expenditure [1–4] – to capture economic and societal determinants of health expenditure, such as income and price developments, the spread of medical technologies and general attitudes towards health care consumption.	OECD 2015
Population	Population is defined as all nationals present in, or temporarily absent from a country, and aliens permanently settled in a country. This indicator shows the number of people that usually live in an area. Growth rates are the annual changes in population resulting from births, deaths and net migration during the year. Total population includes the following: national armed forces stationed abroad; merchant seamen at sea; diplomatic personnel located abroad; civilian aliens resident in the country; displaced persons resident in the country. However, it excludes the following: foreign armed forces stationed in the country; foreign diplomatic personnel located in the country; civilian aliens temporarily in the country. Population projections are a common demographic tool. They provide a basis for other statistical projections, helping governments in their decision making. This indicator is measured in thousands of people. Ageing being an important driver for health expenditure, earlier versions of the draft contained population over 65 (POP65) as the independent variable, producing equivalent results in the regression.	OECD 2015
Deficit ratio	This ratio expresses deficit in terms of GDP	OECD
IMF bailout	This dummy is modeled after Reeves et al. [6] and includes Stand-by Arrangements (SBA) and Extended Fund Facilities (EFF): IMF bailouts include Stand-by Arrangements, usually short-term lending to states to cover the effect of unanticipated shocks, and Extended Fund Facilities, which are usually medium- or long-term lending programs aimed at overcoming weaknesses in the national economy which may have precipitated or exacerbated the shock. IMF becomes 1 the year after the agreement has been made. This reflects the time lag between the loan and the implementation of any changes to government spending, i.e., if an agreement was made in 2008 the variable would measure 1 in 2009 and remain 1 while the bailout is active. When the agreement ends the indicator becomes 0.	IMF’s Monitoring of Fund Arrangements database (MONA)
FR	This dummy variable indicates if a fiscal rule (ER, BBR, RR, DR) is present (1) or not (0) for a given country, in a given year.	IMF 2016
ER	This dummy variable indicates if an expenditure rule is present (1) or not (0), in a given year.	IMF 2016
BBR	This dummy variable indicates if a balanced budget rule is present (1) or not (0), in a given year.	IMF 2016
BBR NAT	This ordinal dummy variable indicates if a balanced budget rule at the national level is present (1) or not (0), in a given year.	IMF 2016
BBR SUPRA	This ordinal dummy variable indicates if a balanced budget rule at the supranational level is present (1) or not (0), in a given year.	IMF 2016
BBR BOTH	This ordinal dummy variable indicates if a balanced budget rule at both the national and supranational level is present (1) or not (0), in a given year.	IMF 2016
DR	This dummy variable indicates if a debt rule is present (1) or not (0), in a given year.	IMF 2016

## Appendix 2

**Table 5:** Fiscal rules targeting public health care expenditure between 1985 and 2014

Country	Expenditure Rule (ER)	Balanced Budget Rule (BBR) National	Balanced Budget Rule (BBR) Supranational	Balanced Budget Rule (BBR) Both
Australia	1985–1988 2009–2014	1985–1988 1998–2014	–	–
Austria^a^	2013-2014	–	1995-1998	1999–2014
Belgium	1993–1998	–	1992–2014	–
Canada	1998–2005	1998–2005	–	–
Chile	–	2001–2014	–	–
Czech Republic	–	–	2004–2014	–
Denmark	1994–2014	–	–	1992–2014
Estonia	–	1993–2003	–	2004–2014
Finland	2003–2014	–	1995–1998	1999–2014
France	1998–2014	–	1992–2012	2013–2014
Germany	1985–2014	1985–1991	–	1992–2014
Greece	2010–2014	–	1992–2014	–
Hungary	2010–2011	–	2012–2014	2004–2011
Iceland	2004–2008	–	–	–
Ireland	–	–	1992–2014	–
Israel	2005–2014	1992–2014	–	–
Italy	–	–	1992–2013	2014
Japan	2006–2008 2010–2014	1985–2014	–	–
Luxembourg	1990–2014	–	1992–2014	–
Mexico	–	2006–2014	–	–
Netherlands	1994–2014	–	1992–2013	2014
New Zealand	–	1994–2014	–	–
Norway	–	2001–2014	–	–
Poland	2011–2014	–	2004–20052008–2014	2006–2007
Portugal	–	–	1992–2014	–
Slovak Republic	–	–	2004–2014	–
Slovenia	–	–	2004–2014	–
Spain	2011–2014	–	1992–2002	2003–2014
Sweden	1997–2014	–	1995–1999	2000–2014
Switzerland^b^	–	–	–	–
United Kingdom	–	–	1992–1996	1997–2014
United States^c^	2011–2014	–	–	–

^a^Modification of the original dataset; Austria introduced a budget cap in 2013, see [37]

^b^Modification of the original dataset; the Swiss fiscal rules (introduced in 2003) cover only the relatively small federal budget and excludes health care expenditure, see [38]

^c^Modification of the original dataset; the 1985 and 1990 ER did not target Medicare or Medicaid spending. The 2011 ER did target Medicare spendingSource: IMF 2015

## Appendix 3

**Table 6:** The association between fiscal rules and the level of public health care expenditure

	Inflation adjusted government health care expenditure in log						
	(1)	(2)	(3)	(4)	(5)	(6)	(7)
	All countries				Exclude Cluster I countries		
FR	− 0.03**				− 0.04***		
	(0.01)				(0.01)		
ER		−0.04**				− 0.05***	
		(0.01)				(0.01)	
BBR (detailed levels)							
National			−0.02				
			(0.02)				
Supranational			−0.06***				
			(0.02)				
Both			−0.08***				
			(0.02)				
BBR				−0.04**			− 0.05***
				(0.01)			(0.01)
Log debt	−0.03**	−0.03*	−0.03*	− 0.03**	−0.03*	− 0.03*	−0.03*
	(0.01)	(0.01)	(0.01)	(0.01)	(0.01)	(0.01)	(0.01)
Log GDP	0.75***	0.71***	0.83***	0.77***	0.68***	0.62***	0.70***
	(0.06)	(0.05)	(0.06)	(0.06)	(0.06)	(0.05)	(0.06)
Log population	0.17	0.22	−0.08	0.13	0.13	0.19	0.08
	(0.12)	(0.12)	(0.15)	(0.12)	(0.13)	(0.13)	(0.13)
IMF bailout	−0.03	− 0.03	− 0.04	− 0.03	− 0.05*	− 0.04	−0.04
	(0.02)	(0.02)	(0.02)	(0.02)	(0.02)	(0.02)	(0.02)
Constant	−12.80***	−12.41***	−11.31***	−12.58***	−10.60***	−9.95***	−10.26***
	(1.64)	(1.62)	(1.71)	(1.62)	(1.73)	(1.71)	(1.71)
*Country FE*	Y	Y	Y	Y	Y	Y	Y
*Year FE*	Y	Y	Y	Y	Y	Y	Y
*N*	717	717	717	717	609	609	609
adj. *R*^2^	0.997	0.997	0.997	0.997	0.997	0.997	0.997

Standard errors are in parentheses

**p* < 0.05, ***p* < 0.01, ****p* < 0.001

## Appendix 4

**Table 7:** The lagged effectiveness of fiscal rules

	Inflation adjusted government health care expenditure in log					
	(1)	(2)	(3)	(4)	(5)	(6)
	Lag: 1 year			Lag: 2 years		
FR	−0.04***			−0.05***		
	(0.01)			(0.01)		
ER		−0.05***			−0.06***	
		(0.01)			(0.01)	
BBR			−0.04***			−0.05***
			(0.01)			(0.01)
Log debt	−0.04**	−0.03*	− 0.04**	− 0.04**	−0.03**	− 0.04**
	(0.01)	(0.01)	(0.01)	(0.01)	(0.01)	(0.01)
Log GDP	0.77***	0.71***	0.77***	0.80***	0.70***	0.78***
	(0.06)	(0.05)	(0.06)	(0.06)	(0.05)	(0.06)
Log population	0.14	0.22	0.11	0.10	0.23	0.08
	(0.12)	(0.12)	(0.12)	(0.12)	(0.12)	(0.12)
IMF bailout	−0.03	−0.03	−0.03	−0.02	−0.03	−0.03
	(0.02)	(0.02)	(0.02)	(0.02)	(0.02)	(0.02)
Constant	−12.82***	−12.35***	−12.43***	−12.77***	−12.22***	−12.24***
	(1.62)	(1.61)	(1.62)	(1.61)	(1.60)	(1.61)
*Country FE*	Y	Y	Y	Y	Y	Y
*Year FE*	Y	Y	Y	Y	Y	Y
*N*	717	717	717	717	717	717
adj. *R*^2^	0.997	0.997	0.997	0.997	0.997	0.997

Standard errors are in parentheses

**p* < 0.05, ***p* < 0.01, ****p* < 0.001

## Appendix 5

**Table 8:** Supporting procedures

	Inflation adjusted government health care expenditure in log				
	(1)	(2)	(3)	(4)	(5)
	ER	BBR		FR	
FR	0.09	−0.03*	−0.02	−0.03**	−0.03*
	(0.05)	(0.01)	(0.01)	(0.01)	(0.01)
FR×FR legal basis level 1	−0.17***	−0.06***			
	(0.04)	(0.02)			
FR×FR legal basis level 2	0.17	0.04*			
	(0.09)	(0.02)			
FR legal basis level 1	0.02				
	(0.07)				
FR legal basis level 1	−0.29**				
	(0.10)				
FR×ceiling			− 0.05***		
			(0.01)		
FR×independent body setting				0.01	
				(0.02)	
FR×independent monitoring					0.06
					(0.06)
Independent monitoring					−0.07
					(0.06)
Log GDP	0.73***	0.75***	0.75***	0.75***	0.76***
	(0.05)	(0.06)	(0.06)	(0.06)	(0.06)
Log population	0.23	0.09	0.19	0.16	0.16
	(0.12)	(0.12)	(0.12)	(0.12)	(0.12)
Log debt	−0.02	−0.02	−0.04**	−0.03**	−0.03**
	(0.01)	(0.01)	(0.01)	(0.01)	(0.01)
Bailout	−0.06*	−0.03	− 0.04	−0.03	− 0.03
	(0.02)	(0.02)	(0.02)	(0.02)	(0.02)
Constant	−12.95***	−11.32***	−12.86***	−12.35***	−12.39***
	(2.02)	(2.00)	(2.00)	(2.02)	(2.02)
*Country FE*	Y	Y	Y	Y	Y
*Year FE*	Y	Y	Y	Y	Y
*N*	717	717	717	717	717
adj. *R*^2^	0.997	0.997	0.997	0.997	0.997

**p* < 0.05, ***p* < 0.01, ****p* < 0.001

## Appendix 6

**Table 9:** The association between public healthcare spending as percentage of GDP (column 1) and various sensitivity checks (columns 2–4)

	Outcome			
	Public healthcare spending as % of GDP	Log public healthcare spending		
	(1)	(2)	(3)	(4)
FR	−0.40***		−0.03*	−0.03*
	(0.07)		(0.01)	(0.01)
Level of DR				
National		−0.03		
		(0.02)		
Supranational		−0.07***		
		(0.02)		
both		−0.07**		
		(0.02)		
Log population	−0.48	− 0.08		0.15
	(0.61)	(0.15)		(0.12)
Log debt	−0.17*	−0.03*	− 0.05***	
	(0.08)	(0.01)	(0.01)	
Log GDP		0.83***	0.77***	0.81***
		(0.06)	(0.05)	(0.06)
Ratio of population 65 and above			0.00	
			(0.00)	
Deficit ratio				−0.01***
				(0.00)
IMF bailout	− 0.09	−0.04	− 0.02	−0.06**
	(0.12)	(0.02)	(0.02)	(0.02)
Constant	16.41	−10.53***	−9.83***	−14.35***
	(10.47)	(2.06)	(1.44)	(1.89)
*Country FE*	Y	Y	Y	Y
*Year FE*	Y	Y	Y	Y
*N*	717	717	735	631

This table presents a series of robustness checks we performed by using similar measures to replace our main model. Column 1 changes the outcome variable from inflation and PPP adjusted public healthcare spending to such spending as a percentage of total GDP. Column 2 replaces the primary variable of interest from general *FR* to *DR*. Columns 3 and 4 replaces *population* and *debt* with ratio of population 65 and above and deficit ratio

Standard errors in parentheses **p* < 0.05, ***p* < 0.01, ****p* < 0.001

## Appendix 7

**Table 10:** The association between fiscal rules and average length of stay in a curative care facility

	Average length of stay		
	(1)	(2)	(3)
FR	1.68***		
	(0.24)		
ER		−0.20	
		(0.25)	
BBR			2.16***
			(0.24)
Log GDP	2.00*	3.93***	1.44
	(1.00)	(1.00)	(0.98)
Log population (mil)	6.79**	5.46*	8.57***
	(2.19)	(2.27)	(2.15)
Log debt	−2.06***	−2.03***	−2.02***
	(0.24)	(0.25)	(0.23)
Bailout	0.60	0.53	0.49
	(0.37)	(0.39)	(0.36)
Constant	0.24	−47.34	9.75
	(25.56)	(25.68)	(24.83)
*N*	630	630	630
adj. *R*^2^	0.962	0.959	0.964

Standard errors in parentheses

**p* < 0.05, ***p* < 0.01, ****p* < 0.001

## Abbreviations

BBR: Balanced budget rule
DR: Debt rule
ER: Expenditure rule
FR: Fiscal rule
GDP: Gross domestic product
IMF: International monetary fund
OECD: Organisation for economic co-operation and development
SGP: Stability and growth pact

## Footnotes

1 ERs set limits on total, primary, or current spending; RRs set ceilings or floors on revenues and are aimed at boosting revenue collection and/or preventing an excessive tax burden; DRs set an explicit limit or target for public debt in percent of GDP; BBRs constrain the variable that primarily influences the debt ratio and are largely under the control of policy makers. (source: Budina et al. [5]).

2 In the ‘corrective arm’ of the SGP, the Excessive Deficit Procedure (EDP) ensures the correction of excessive budget deficits or excessive public debt levels. It is a step-by-step approach for reining in excessive deficits and reducing excessive debts. The EU Treaty defines an excessive budget deficit as one greater than 3% of GDP. Public debt is considered excessive under the Treaty if it exceeds 60% of GDP without diminishing at an adequate rate (defined as a decrease of the excess debt by 5% per year on average over three years) (source: ec.europa.eu)

3 Government healthcare expenditure, GDP, debt and population are expressed in natural log so that we are able to interpret the results in percentage change. Expenditure, GDP and debt are also measured in international dollars (i.e. purchasing power parity) so that we are able to compare different countries across time.

4 It should be noted that the analysis of Wendt only includes fifteen European countries and that some countries are characterized as social insurance systems (such as the Netherlands), but do not fit Wendt’s typology.

5 A delay in reporting of 6 months up to two years is not uncommon throughout the OECD (see for an overview of reporting delays per country OECD 2015, 92).
